# A Lower Bound on the Estimation Variance of Direction-of-Arrival and Skew Angle of a Biaxial Velocity Sensor Suffering from Stochastic Loss of Perpendicularity

**DOI:** 10.3390/s22218464

**Published:** 2022-11-03

**Authors:** Chibuzo Joseph Nnonyelu, Meng Jiang, Jan Lundgren

**Affiliations:** Sensible Things that Communicate Research Centre, Mid Sweden University, 852 30 Sundsvall, Sweden

**Keywords:** Cramér-Rao bound, biaxial sensor, direction-of-arrival, u-u probe, sensor perturbation

## Abstract

The biaxial velocity sensor comprises two nominally perpendicular particle velocity sensors and a collocated pressure sensor. Due to real-world imperfections in manufacturing or setup errors, the two axes may suffer from perpendicularity losses. To analytically study how skewness affects its direction-finding performance, the hybrid Cramér-Rao bound (HCRB) of the directions-of-arrival for the polar angle, azimuth angle and the skew angle of a biaxial velocity sensor that suffers from stochastic loss of perpendicularity were derived in closed form. The skew angle was modeled as a zero-mean Gaussian random variable of a known variance, which was assumed to be very small, to capture the uncertainty in the orthogonality of the biaxial velocity sensor. The analysis shows that for the polar and azimuth angle, the loss of perpendicularity introduces the variation of the HCRB along the azimuth angle axis, which is independent of the skew angle, but on its variance. The dynamic range of this variation increases as the variance of the skew angle increases. For the estimation of the skew angle, the HCRB of the skew angle is bounded upwards by the variance of the skew angle and varies with the azimuth angle. The hybrid maximum likelihood- maximum a posterior (hybrid ML/MAP) estimator was used to verify the derived bounds.

## 1. Introduction

Research on signal processing involving acoustic particle velocity sensing has been an important part of the field of acoustics sensing throughout the decades. The study of the performance of acoustic particle velocity sensors regarding direction finding could be traced back to 1994 in [1], as well as research focusing on moving source tracking [2], acoustic communication [3,4], feature extraction of underwater emitter [5], geoacoustic inversion problems [6] and acoustic shielding and focusing [7]. The biaxial velocity sensor consists of two collocated (but orthogonal uniaxial) acoustic particle velocity sensors that individually measure acoustic particle velocities along their main axes and the collocated pressure sensor at the origin. The biaxial velocity sensor was implemented in [8,9,10]. This spatial arrangement offers an array that is independent of the frequency of the emitter or sensor displacement. That is, the two component-sensor spatial collocations intrinsically decouple the time–frequency dimensions from the azimuth–elevation spatial dimensions of the data. The use of the biaxial velocity sensor for direction finding can be found in [11,12,13]. Moreover, in beamforming applications, its directivity was studied in [14,15].

Due to real-world imperfections of the manufacturing process, as well as set-up errors, the biaxial velocity sensor may become non-orthogonal, introducing estimation errors when used in direction-finding applications. In beamforming applications, the shape of the spatial-matched-filter beam pattern of a non-orthogonal biaxial velocity sensor has been shown to remain the same with an analytically derived pointing bias [15,16]. Such a phenomenon was also demonstrated for a triaxial velocity sensor suffering from non-perpendicularity in one axis [17].

The directions of arrival estimation performances of perturbed orthogonal vector sensors have been studied in [13,18,19]. In [13], an algorithm was developed for direction finding using an imperfect (non-orthogonal) biaxial velocity sensor. This study did not derive a theoretical bound of the performance of such an imperfect biaxial vector sensor. Moreover, the skew angle was assumed to be deterministic. In [19], the Cramér-Rao bound of an acoustic vector sensor suffering from nonideal gain–phase responses, non-collocation, or non-orthogonal orientation was studied. In this study, the skew angle was considered deterministic, which does not reflect a real-world situation, as a randomly selected biaxial sensor suffers from skewness. Moreover, this skewness was not uniform across the sensors. De Freitas [18] proposed a method for estimating the misalignment angle and vector fidelity of a nominally triaxial accelerometer. This analysis can easily be extended to the biaxial acoustic vector sensor. This study, however, did not study the performance of such a misaligned vector sensor. In general, these studies modeled the skew angle as an unknown deterministic parameter. This assumption, as done by [13,19], is less practical since skew angles vary across sensors (making prior knowledge of the skew angle distribution an important feature to consider). This becomes more useful for an array of multiple biaxial velocity sensors. Therefore, modeling the skew angle as stochastic with the right prior is important and offers great engineering and practical insights on how the uncertainty in the skewness affects the direction-finding performance.

In the direction of arrival estimation, the directions of incident sound sources (at an array) are estimated from a series of measurements taken at the array. The performance of an unbiased estimator can be assessed by the lower bound on the mean square estimation error. The Cramér-Rao bound (CRB), since its introduction in the mid-1940s, has been a widely used lower bound on the mean square estimation error of unbiased estimates of parameter vectors [20]. This is attributed to the asymptotic theorem that attests to its attainability by the maximum-likelihood estimator in the large sample regime. Hence, it is often used as a performance reference of a given algorithm or as a tool to analyze inherent limitations in the problem [21]. Other bounds on the mean square estimation error of unbiased estimators have been proposed and compared to the CRB in terms of tightness and attainability by estimators [22,23].

For the lower bound of the mean square estimation error of random parameters, the Bayesian CRB [24], the Bobrovsky–Zakai bound [25], and others apply. In cases where the parameter vector contained both random and deterministic parameters, the hybrid Cramér-Rao (HCRB) bound was firstly derived under the assumption that the marginal probability density function of the random parameter was independent of the deterministic parameters [26]. This condition was later relaxed in [27]. Other bounds exist in the literature: the modified Cramér-Rao bound (MCRB) [28], which converges to the HCRB for large amounts of data or a high signal-to-noise ratio, the Miller–Chang CRB-type bound [29], and other modified versions of the CRB [30,31]. While the Miller–Chang is obtained by taking the expectation after the inversion of the Fisher information matrix, the MCRB takes the expectation just before the inversion. These two bonds give the lower bond for just the deterministic unknown parameters while treating the random parameters as nuisance parameters. Some literature studies have studied the closeness, tightness, and regularity conditions of these modified CRBs [32,33,34,35,36].

In the estimation problem studied in this paper, similar to the angle-of-arrival, the skew angle does not vary while the data are measured but any randomly selected biaxial velocity sensor suffers from non-orthogonality. In this case, the skew angle is not known to the estimator but is assumed to follow the Gaussian distribution with a mean of zero and a known variance that is far less than a given positive real number. This presumption on the variance helps to render the probability of skew angles far greater than zero as negligible; a reasonable presumption for any well-built sensor for practical use [37]. This variance can be specified by the equipment manufacturer based on a series of measurements and on their manufacturing processes. The lower bound of the mean square error (MSE) of such an estimation problem can be studied under the non-standard deterministic estimation summarized in [27,33], if the estimation of the skew angle is not of interest. However, given that the estimation of the skew angle is of interest in the problem at hand, the hybrid Cramer–Rao bound gives the lower bounds of the MSE of the joint estimation of the direction of arrival and the skew angle given the regularity conditions [26]. Therefore, in this paper, the hybrid Cramér-Rao bound (HCRB) of the emitter’s polar and azimuth angle-of-arrival, and the skew angle of the sensors, were derived in closed form and analyzed. The maximum a posterior estimator was used to verify the derived bounds for the polar and azimuth. This analysis differs from [13], where aside from not deriving the Cramér-Rao bound, the authors assumed the skew angle to be deterministic and unknown (see also [38]), not considering the effect of the uncertainty in the measurement on the direction-finding performance of the skewed biaxial sensor.

The rest of the paper is organized as follows: The statistical data model is presented in Section 2 where the array manifold of the non-orthogonal biaxial velocity sensor was derived and the received signal model is described. The hybrid Cramér-Rao bound is presented in closed form in Section 3 and the derived bounds are discussed in Section 4. The derived HCRBs are verified using the joint/hybrid MAP and ML estimator (JMAPMLE) in Section 5. Finally, the study is concluded in Section 6.

## 2. Statistical Data Model

### 2.1. Array Manifold

The single velocity sensor’s “figure-8 gain” pattern is mathematically described as a=cos(φ) where φ∈[0,2π) is the angle that the incident wave makes with the main axis of the sensor. For the collocated perfectly orthogonal biaxial velocity sensor pair with a pressure sensor, its array manifold

(1) $$\begin{array}{ccc} a(\theta ,\phi ) & = & \left[\begin{array}{c} \cos(\phi )\sin(\theta )\\ \sin(\phi )\sin(\theta )\\ 1 \end{array}\right] \end{array}$$

where θ∈[0,π] is the polar angle and ϕ∈[0,2π) is the azimuth angle of the incident source. The first entry is the response of the velocity sensor aligned with the positive nominal *x*-axis, the second entry represents the response of the velocity sensor aligned with the positive *y*-axis, and the third entry is the response of the pressure sensor.

**Figure 1:**
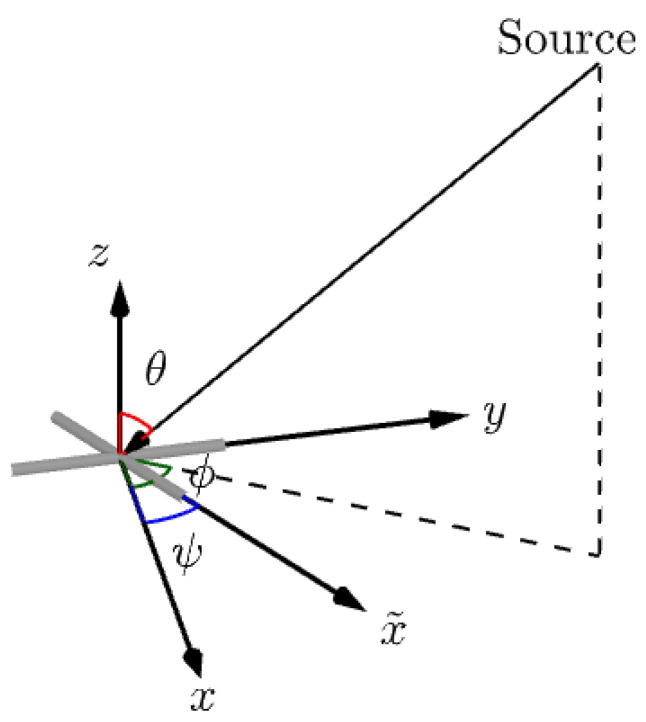
Diagrammatic representation of the skewed biaxial velocity sensor.

Without loss of generality, the *y*-axis is assumed to perfectly align with the nominal *y*-axis while the *x*-axis deviates from the nominal *x*-axis by an angle ψ to $\tilde{x}$-axis, as shown in Figure 1. This orthogonality perturbation is captured in the matrix

(2) $$\begin{array}{ccc} R(\psi ) & = & \left[\begin{array}{ccc} \cos\psi & -\sin\psi & 0\\ 0 & 1 & 0\\ 0 & 0 & 1 \end{array}\right] \end{array}$$

where the angle ψ is stochastic and follows a zero-mean Gaussian distribution with variance $\sigma_{\psi }^{2}$, i.e., $\psi ∼N(0,\sigma_{\psi }^{2})$. Therefore, the array manifold of such a non-orthogonal biaxial velocity sensor is given as

(3) $$\begin{array}{ccc} {\tilde{a}}_{\psi }\left(\theta ,\phi \right) & = & R\;a(\theta ,\phi )\\ & = & \left[\begin{array}{c} \cos(\phi +\psi )\sin(\theta )\\ \sin(\phi )\sin(\theta )\\ 1 \end{array}\right]. \end{array}$$

### 2.2. Received Signal Model

The array will receive the complex 3×1 data at discrete time instant *k*,

(4) $$\begin{array}{ccc} z(k) & = & s\left(k\right){\tilde{a}}_{\psi }\left(\theta ,\phi \right)+n\left(k\right),\;k=1,2,\cdots ,K, \end{array}$$

where *K* is the total number of time snapshots. The incident signal is assumed to be pure tone complex exponential $s\left(k\right)=\sigma_{s}e^{j(\omega t+\varphi )}$, where $\sigma_{s}$, ω, and φ∈[−π,π] are the a priori known signal amplitude, angular frequency, and phase, respectively. The pure tone complex exponential is chosen to maintain focus on the uncertainty of the direction parameter estimation since narrow-band signals can be reduced to combinations of sine waves with known amplitudes and phases using the discrete Fourier transform.

The noise at each sensor was assumed to be independent and an identically distributed–spatiotemporally uncorrelated circularly-symmetric zero-mean white complex Gaussian random process with a priori known variance $\sigma_{n}^{2}$, i.e., ${\left[n\right]}_{\ell }∼\mathrm{CN}\left(0,\sigma_{n}^{2}\right)$ and also independent of the source, where ℓ=1,2,3 denotes the *ℓ*th entry of vector n. Indeed, $\sigma_{n}^{2}$ can be measured by turning off the sensors.

## 3. Deriving the Hybrid Cramér-Rao Bound

For multiple snapshots, the received data are arranged as the 3K×1 vector

(5) $$\begin{array}{c} \;\tilde{z}={\left[\begin{array}{cccc} z{\left(1\right)}^{T} & z{\left(2\right)}^{T} & \cdots & z{\left(K\right)}^{T} \end{array}\right]}^{T},\\ \equiv s⊗{\tilde{a}}_{\psi }\left(\theta ,\phi \right)+\tilde{n}\;, \end{array}$$

where $s={\left[\begin{array}{cccc} s(1) & s(2) & \cdots & s(K) \end{array}\right]}^{T}$ and $\tilde{n}={\left[\begin{array}{cccc} n{\left(1\right)}^{T} & n{\left(2\right)}^{T} & \cdots & n{\left(K\right)}^{T} \end{array}\right]}^{T}$.

The received data $\tilde{z}$ are parameterized by ψ, and the conditional data have the means

(6) $$\begin{array}{ccc} \mu & = & E\left[\tilde{z}|\psi \right]\;\;=\;\;E\left[s⊗{\tilde{a}}_{\psi }\right]\;\;=\;\;s⊗{\tilde{a}}_{\psi }, \end{array}$$

and a covariance matrix

(7) $$\begin{array}{c} \;\Gamma =E\left[\left(\tilde{z}-\mu \right){\left(\tilde{z}-\mu \right)}^{H}|\psi \right]\\ =\sigma_{n}^{2}I_{3K}, \end{array}$$

where $I_{3K}$ is an identity matrix of order 3K. Since this paper focuses on the adverse effects of the skew angle on polar and azimuth angles estimation, a simple data model will be used to avoid unnecessary mathematical complexities that distract from the above focus. More specifically, we assume that the signal amplitude s is known or has been estimated prior to the DoA estimation. Thus, only three unknown parameters of interest remain, collected here as a vector

(8) $$\begin{array}{ccc} \xi & = & {\left[\begin{array}{ccc} \theta & \phi & \psi \end{array}\right]}^{T}. \end{array}$$

The hybrid Cramér-Rao bound is the inverse of the hybrid Fisher information matrix [26,39]

(9) $$\begin{array}{c} \;J=\left[\begin{array}{ccc} J_{\theta ,\theta } & J_{\theta ,\phi } & J_{\theta ,\psi }\\ J_{\theta ,\phi } & J_{\phi ,\phi } & J_{\phi ,\psi }\\ J_{\theta ,\psi } & J_{\phi ,\psi } & J_{\psi ,\psi } \end{array}\right]\\ =E_{\psi }\left[H(\xi )\right]+\left[\begin{array}{cc} 0_{2\times 2} & 0_{2\times 1}\\ 0_{1\times 2} & \Gamma_{\psi }^{-1} \end{array}\right], \end{array}$$

where $0_{L\times M}$ is an L×M matrix of zeroes and $\Gamma_{\psi }^{-1}=\sigma_{v}^{-2}$ (inverse of the covariance of the skew angle). The (i,j)th entry of the matrix H(ξ) is given as

(10) [H(ξ)]i,j=2Re∂μH∂ξiΓ−1∂μ∂ξj+TrΓ−1∂Γ∂ξiΓ−1∂Γ∂ξj=2σn2Re∂μH∂ξi∂μ∂ξj=2σn2ResH⊗∂a˜H∂ξis⊗∂a˜∂ξj=2sHsσn2∂a˜H∂ξi∂a˜∂ξj=2Kσs2σn2∂a˜H∂ξi∂a˜∂ξj

where $\xi_{i}$ and $\xi_{j}$ are the *i*th and *j*th entries of vector ξ, respectively, and Tr(·) denotes the trace of a matrix. The trace term disappeared since the covariance matrix is independent of all the unknown parameters. Note also that $s^{H}s=K\sigma_{s}^{2}$.

Toward obtaining the hybrid Fisher information matrix,

(11) $$\begin{array}{ccc} \frac{\partial \tilde{a}}{\partial \theta } & = & \left[\begin{array}{c} \cos(\phi +\psi )\cos(\theta )\\ \sin(\phi )\cos(\theta )\\ 0 \end{array}\right], \end{array}$$

(12) $$\begin{array}{ccc} \frac{\partial \tilde{a}}{\partial \phi } & = & \left[\begin{array}{c} -\sin(\phi +\psi )\sin(\theta )\\ \cos(\phi )\sin(\theta )\\ 0 \end{array}\right], \end{array}$$

(13) $$\begin{array}{ccc} \frac{\partial \tilde{a}}{\partial \psi } & = & \left[\begin{array}{c} -\sin(\phi +\psi )\sin(\theta )\\ 0\\ 0 \end{array}\right]. \end{array}$$

Hence,

(14) $$\begin{array}{ccc} H_{\theta ,\theta } & = & 2K\frac{\sigma_{s}^{2}}{\sigma_{n}^{2}}\left[\cos^{2}\left(\phi +\psi \right)+\sin^{2}\left(\phi \right)\right]\cos^{2}\theta , \end{array}$$

(15) $$\begin{array}{ccc} H_{\theta ,\phi } & = & \frac{K}{2}\frac{\sigma_{s}^{2}}{\sigma_{n}^{2}}\left[\sin(2\phi )-\sin(2\phi +2\psi )\right]\sin\left(2\theta \right), \end{array}$$

(16) $$\begin{array}{ccc} H_{\theta ,\psi } & = & -\frac{K}{2}\frac{\sigma_{s}^{2}}{\sigma_{n}^{2}}\sin\left(2\phi +2\psi \right)\sin\left(2\theta \right), \end{array}$$

(17) $$\begin{array}{ccc} H_{\phi ,\phi } & = & 2K\frac{\sigma_{s}^{2}}{\sigma_{n}^{2}}\left[\sin^{2}\left(\phi +\psi \right)+\cos^{2}\left(\phi \right)\right]\sin^{2}\theta , \end{array}$$

(18) $$\begin{array}{ccc} H_{\phi ,\theta } & = & H_{\theta ,\phi }, \end{array}$$

(19) $$\begin{array}{ccc} H_{\phi ,\psi } & = & 2K\frac{\sigma_{s}^{2}}{\sigma_{n}^{2}}\sin^{2}\left(\phi +\psi \right)\sin^{2}\theta , \end{array}$$

(20) $$\begin{array}{ccc} H_{\psi ,\psi } & = & H_{\psi ,\phi }\;\;=\;\;H_{\phi ,\psi }, \end{array}$$

(21) $$\begin{array}{ccc} H_{\psi ,\theta } & = & H_{\theta ,\psi }. \end{array}$$

Before proceeding, the following propositions are made for constant ϕ:

1. If $\psi ∼N(0,\sigma_{\psi }^{2})$, then $\left(\psi +\phi \right)∼N\left(\phi ,\sigma_{\psi }^{2}\right)$ and $\left(2\psi +2\phi \right)∼N(2\phi ,{\left(2\sigma_{v}\right)}^{2})$.
2. If $\left(\phi +\psi \right)∼N\left(\phi ,\sigma_{\psi }^{2}\right)$, then $E\left[\cos\left(\psi +\phi \right)\right]=e^{-\sigma_{\psi }^{2}/2}\cos\phi$ and $E\left[\sin\left(\psi +\phi \right)\right]=e^{-\sigma_{\psi }^{2}/2}\sin\phi$ (please see Appendix A for the proof).
3. If $\left(2\phi +2\psi \right)∼N(2\phi ,{\left(2\sigma_{v}\right)}^{2})$, then $E\left[\sin\left(2\phi +2\psi \right)\right]=e^{-2\sigma_{\psi }^{2}}\sin\left(2\phi \right)$ and $E\left[\cos\left(2\phi +2\psi \right)\right]=e^{-2\sigma_{\psi }^{2}}\cos\left(2\phi \right)$.

The above propositions are used to evaluate the expectations with respect to ψ subsequently. The entries of the hybrid Fisher information matrix are derived as

(22) $$\begin{array}{ccc} J_{\theta ,\theta } & = & E_{\psi }\left[H_{\theta ,\theta }\right]\\ & = & K\frac{\sigma_{s}^{2}}{\sigma_{n}^{2}}\left[2-\left(1-e^{-2\sigma_{\psi }^{2}}\right)\cos\left(2\phi \right)\right]\cos^{2}\theta , \end{array}$$

(23) $$\begin{array}{ccc} J_{\theta ,\phi } & = & E_{\psi }\left[H_{\theta ,\phi }\right]\\ & = & \frac{K}{2}\frac{\sigma_{s}^{2}}{\sigma_{n}^{2}}\left(1-e^{-2\sigma_{\psi }^{2}}\right)\sin\left(2\phi \right)\sin\left(2\theta \right), \end{array}$$

(24) $$\begin{array}{ccc} J_{\theta ,\psi } & = & E_{\psi }\left[H_{\theta ,\psi }\right]\\ & = & -\frac{K}{2}\frac{\sigma_{s}^{2}}{\sigma_{n}^{2}}e^{-2\sigma_{\psi }^{2}}\sin\left(2\phi \right)\sin\left(2\theta \right), \end{array}$$

(25) $$\begin{array}{ccc} J_{\phi ,\phi } & = & E_{\psi }\left[H_{\phi ,\phi }\right]\\ & = & K\frac{\sigma_{s}^{2}}{\sigma_{n}^{2}}\left[2+\left(1-e^{-2\sigma_{\psi }^{2}}\right)\cos\left(2\phi \right)\right]\sin^{2}\theta , \end{array}$$

(26) $$\begin{array}{ccc} J_{\phi ,\theta } & = & J_{\theta ,\phi }, \end{array}$$

(27) $$\begin{array}{ccc} J_{\phi ,\psi } & = & E_{\psi }\left[H_{\phi ,\psi }\right]\\ & = & K\frac{\sigma_{s}^{2}}{\sigma_{n}^{2}}\left(1-e^{-2\sigma_{\psi }^{2}}\cos\left(2\phi \right)\right)\sin^{2}\theta , \end{array}$$

(28) $$\begin{array}{ccc} J_{\psi ,\psi } & = & E_{\psi }\left[H_{\psi ,\psi }\right]+\sigma_{v}^{-2}\\ & = & K\frac{\sigma_{s}^{2}}{\sigma_{n}^{2}}\left(1-e^{-2\sigma_{\psi }^{2}}\cos\left(2\phi \right)\right)\sin^{2}\theta +\sigma_{v}^{-2}, \end{array}$$

(29) $$\begin{array}{ccc} J_{\psi ,\theta } & = & J_{\theta ,\psi }, \end{array}$$

(30) $$\begin{array}{ccc} J_{\psi ,\phi } & = & J_{\phi ,\psi }. \end{array}$$

The hybrid Cramer–Rao bounds of the polar angle, azimuth angle, and skew angle are the diagonal entries of the $J^{-1}$, given ${\left[J^{-1}\right]}_{1,1}$ in (31), ${\left[J^{-1}\right]}_{2,2}$ in (32), and ${\left[J^{-1}\right]}_{3,3}$ in (33), respectively.

(31) $$\begin{array}{c} {\mathrm{HCRB}}_{\theta }\;\;=\\ \frac{2K\sigma_{\psi }^{2}\left(\frac{\sigma_{s}^{2}}{\sigma_{n}^{2}}\right)\cos^{2}\left(\phi \right)\tan^{2}\left(\theta \right)\left[e^{-2\sigma_{\psi }^{2}}\cos\left(2\phi \right)-1\right]-\sec^{2}\left(\theta \right)\left[2-\left(e^{-2\sigma_{\psi }^{2}}-1\right)\cos\left(2\phi \right)\right]}{K\left(\frac{\sigma_{s}^{2}}{\sigma_{n}^{2}}\right)\left(e^{-2\sigma_{\psi }^{2}}+1\right)\left[\left(e^{-2\sigma_{\psi }^{2}}-3\right)+2K\sigma_{\psi }^{2}\left(\frac{\sigma_{s}^{2}}{\sigma_{n}^{2}}\right)\left(e^{-2\sigma_{\psi }^{2}}-1\right)\cos^{2}\left(\phi \right)\sin^{2}\left(\theta \right)\right]} \end{array}$$

where sec(θ)=1/cos(θ).

(32) $$\begin{array}{c} {\mathrm{HCRB}}_{\phi }\;\;=\\ \frac{K\sigma_{\psi }^{2}\left(\frac{\sigma_{s}^{2}}{\sigma_{n}^{2}}\right)\left(e^{-4\sigma_{\psi }^{2}}-2+\left[1+2e^{-2\sigma_{\psi }^{2}}\sin^{2}\left(\phi \right)\right]\cos\left(2\phi \right)\right)-\csc^{2}\left(\theta \right)\left[2+\left(e^{-2\sigma_{\psi }^{2}}-1\right)\cos\left(2\phi \right)\right]}{K\left(\frac{\sigma_{s}^{2}}{\sigma_{n}^{2}}\right)\left(e^{-2\sigma_{\psi }^{2}}+1\right)\left[\left(e^{-2\sigma_{\psi }^{2}}-3\right)+2K\sigma_{\psi }^{2}\left(\frac{\sigma_{s}^{2}}{\sigma_{n}^{2}}\right)\left(e^{-2\sigma_{\psi }^{2}}-1\right)\cos^{2}\left(\phi \right)\sin^{2}\left(\theta \right)\right]} \end{array}$$

(33) $$\begin{array}{ccc} {\mathrm{HCRB}}_{\psi } & = & \frac{\sigma_{\psi }^{2}\left(e^{-2\sigma_{\psi }^{2}}-3\right)}{\left(e^{-2\sigma_{\psi }^{2}}-3\right)+2K\left(\sigma_{s}^{2}/\sigma_{n}^{2}\right)\sigma_{\psi }^{2}\left(e^{-2\sigma_{\psi }^{2}}-1\right)\cos^{2}\left(\phi \right)\sin^{2}\left(\theta \right)}. \end{array}$$

## 4. Discussing the Derived Bounds

The hybrid Cramér-Rao lower bounds for the polar angle, azimuth angle, and skew angle are presented in Section 3. The derived bounds will be discussed in this section.

### 4.1. Hybrid CRB for Polar Angle, HCRB${}_{\theta }$

To understand how the skewness affects the performance, we first look at the perfectly orthogonal biaxial velocity sensor. By setting $\sigma_{v}=0$, (31) reduces to

(34) $$\begin{array}{ccc} {\left.{\mathrm{HCRB}}_{\theta }\right|}_{\sigma_{\psi }=0} & = & \frac{\sec^{2}\theta }{2K(\sigma_{s}^{2}/\sigma_{n}^{2})}. \end{array}$$

**Figure 2:**
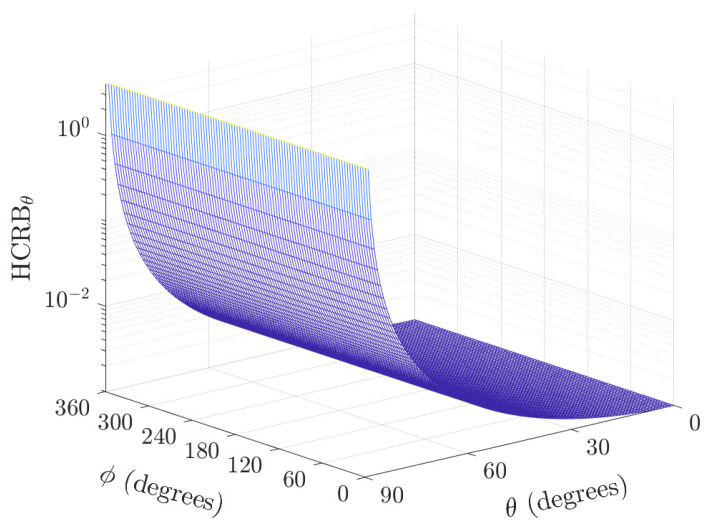
Plot of ${\mathrm{HCRB}}_{\theta }$ versus the direction of the arrival polar and azimuth angles for K=100 samples, $\sigma_{s}^{2}/\sigma_{n}^{2}=5$, and $\sigma_{v}=0$ (a perfectly perpendicular case).

As expected, the ${\mathrm{HCRB}}_{\theta }$ is independent of the azimuth angle for the perpendicular biaxial velocity sensor pair (i.e., $\sigma_{\psi }^{2}=0$). Moreover, the lowest bound is obtained at $\theta =0^{\circ }$ (see Figure 2).

**Figure 3:**
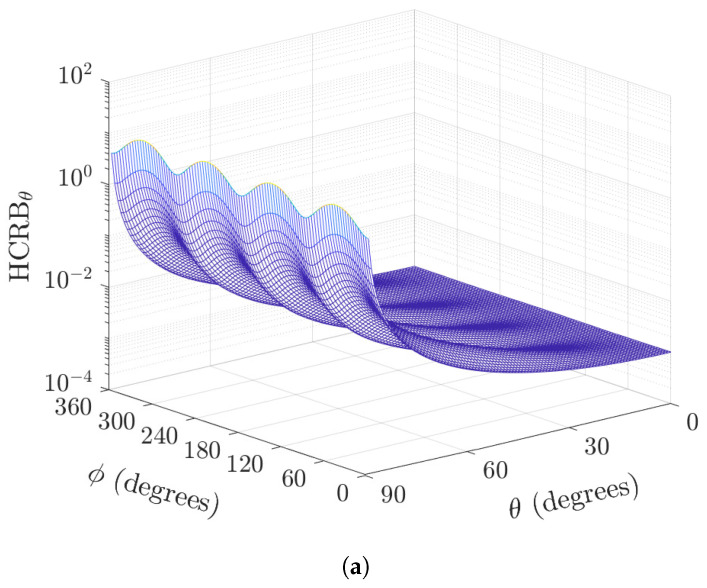

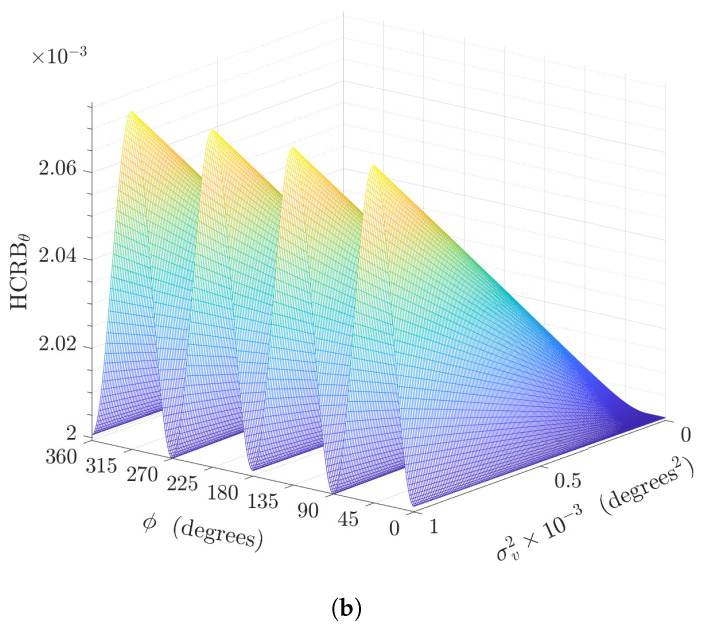
Plot of ${\mathrm{HCRB}}_{\theta }$ versus the directions-of-arrival for the azimuth angles and (**a**) polar angle $\theta \in [0,90^{\circ }]$ ($\sigma_{v}=0.0873$ radian), and (**b**) skew angle variance $\sigma_{\psi }^{2}\in \left[0,1^{\circ }\right]$ ($\theta =45^{\circ }$), both for K=100 samples, $\sigma_{s}^{2}/\sigma_{n}^{2}=5$.

For the non-orthogonal biaxial vector sensor, the skewness introduces variations in the performance along the azimuth angle, as shown in Figure 3. This variation is greater at $\theta =90^{\circ }$ (please see Figure 3a). Moreover, for a given $\theta \in (0,90^{\circ })$, the increase in skew angle variance results in increased variation of the HCRB (please see Figure 3b).

**Figure 4:**
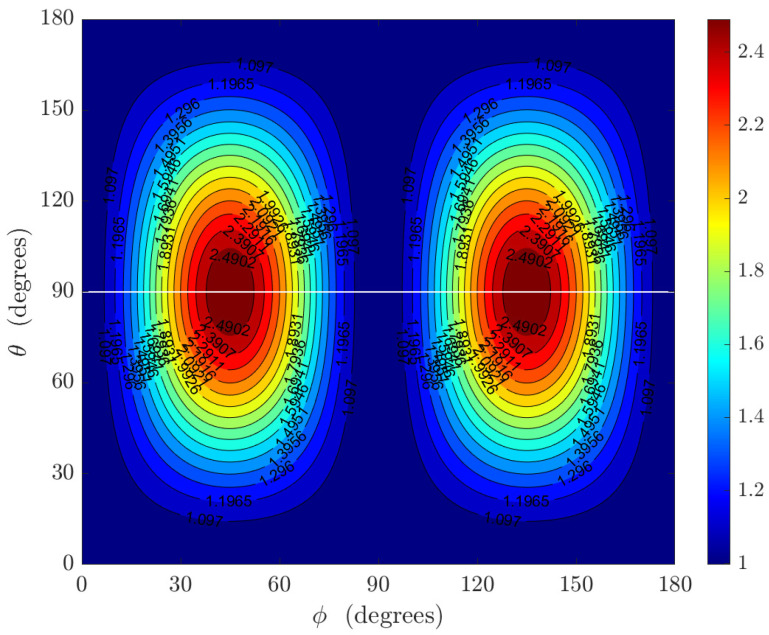
Plot of the performance ratio $r_{\theta }$ versus the polar angle and azimuth angle for $\sigma_{\psi }=0.05$.

For more insight, we look at the ratio of the HCRB of the skewed biaxial vector sensor to the perfectly orthogonal case. We define the performance ratio

(35) $$\begin{array}{ccc} r_{\theta } & := & \frac{{\mathrm{HRCB}}_{\theta }}{{\left.{\mathrm{HCRB}}_{\theta }\right|}_{\sigma_{\psi }=0}} \end{array}$$

such that $r_{\theta }>1$ indicates the degraded performance, $r_{\theta }<1$ implies the improved performance, and $r_{\theta }=1$ indicates equal performance. The plot of the performance ratio $r_{\theta }$ versus the direction of arrival is shown in Figure 4.

The maximum degradation occurs every 90${}^{\circ }$ starting from 45${}^{\circ }$. This trend is also noticed in Figure 3b, where the bound for the polar angle is maximum at $45^{\circ }+n90^{\circ }$, n=0,1,2,3. In Figure 4, where $\sigma_{\psi }=0.05$, a maximum performance ratio of approximately 2.5 is obtained. A slight performance improvement occurs at $\phi =90^{\circ }$ where the minimum performance ratio of 0.7 occurs for $\sigma_{\psi }=1$. This minimum performance ratio increases as $\sigma_{\psi }$ decreases.

### 4.2. Hybrid CRB for Azimuth Angle, HCRB${}_{\phi }$

The hybrid CRB for the azimuth angle is derived as (32). For a proper understanding of how the skewness affects the performance, we first look at the perfectly orthogonal biaxial velocity sensor. By setting $\sigma_{v}=0$, (32) reduces to

(36) $$\begin{array}{ccc} {\left.{\mathrm{HCRB}}_{\phi }\right|}_{\sigma_{v}=0} & = & \frac{\csc^{2}\theta }{2K(\sigma_{s}^{2}/\sigma_{n}^{2})}. \end{array}$$

**Figure 5:**
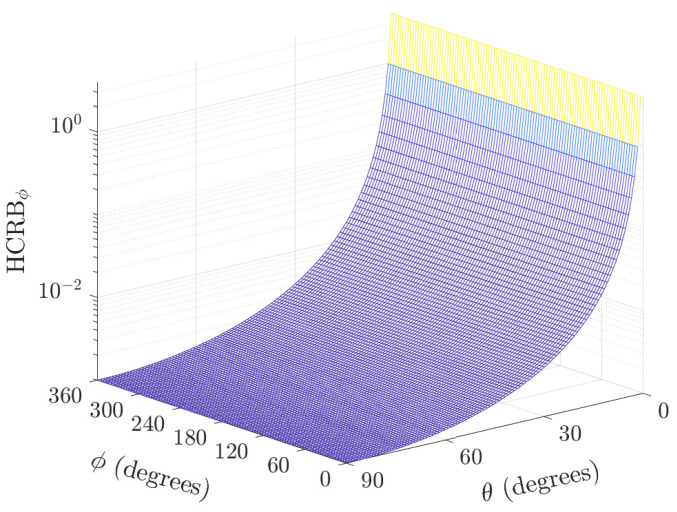
Plot of ${\mathrm{HCRB}}_{\phi }$ versus direction of arrival polar and azimuth angles for K=100 samples, $\sigma_{s}^{2}/\sigma_{n}^{2}=5$, and $\sigma_{v}=0$ (a perfectly perpendicular case).

Figure 5 shows the ${\mathrm{HCRB}}_{\phi }$ for a case of $\sigma_{v}=0$, i.e., a perfectly perpendicular biaxial velocity sensor. The least ${\mathrm{HCRB}}_{\phi }$ occurs at $\theta =90^{\circ }$. Moreover, ${\mathrm{HCRB}}_{\phi }$ does not vary with azimuth angle ϕ.

**Figure 6:**
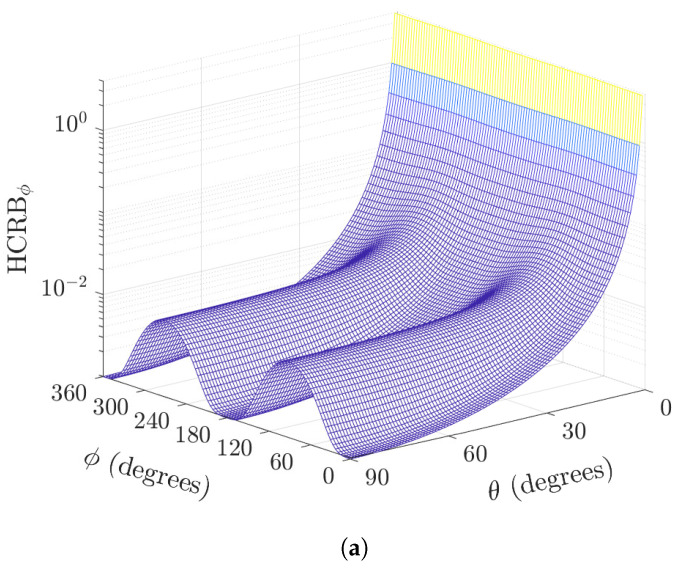

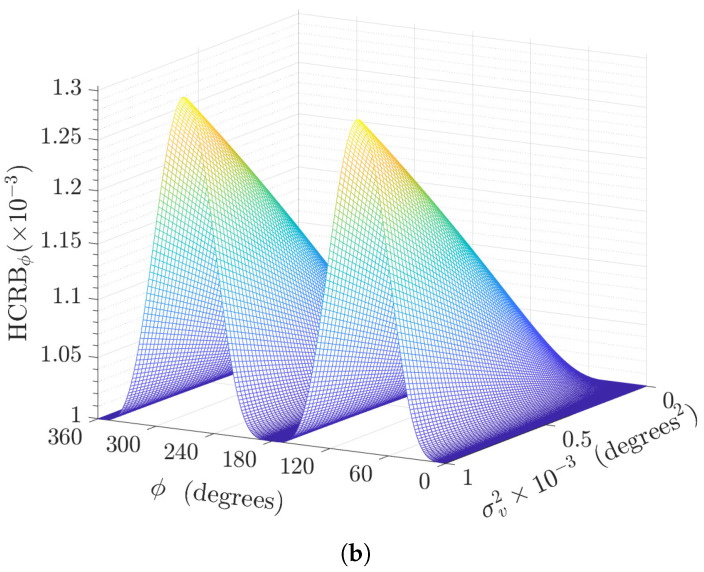
Plot of ${\mathrm{HCRB}}_{\phi }$ versus the directions-of-arrival for the azimuth angles and (**a**) polar angle $\theta \in [0,90^{\circ }]$ ($\sigma_{v}=0.0873$ radian), and (**b**) skew angle variance $\sigma_{\psi }^{2}\in \left[0,1^{\circ }\right]$, both for K=100 samples, $\sigma_{s}^{2}/\sigma_{n}^{2}=5$.

For the non-orthogonal case, the skewness introduces variation in ${\mathrm{HCRB}}_{\phi }$ along the azimuth angle as shown in Figure 6. This variation is greater at $\theta =90^{\circ }$ and more so at $\phi =90^{\circ }$ and $270^{\circ }$ (please see Figure 6a). Moreover, for a given $\theta \in (0,90^{\circ })$, the increase in skew angle variance results in an increased variation of the HCRB (please see Figure 6b) for $\theta =90^{\circ }$.

**Figure 7:**
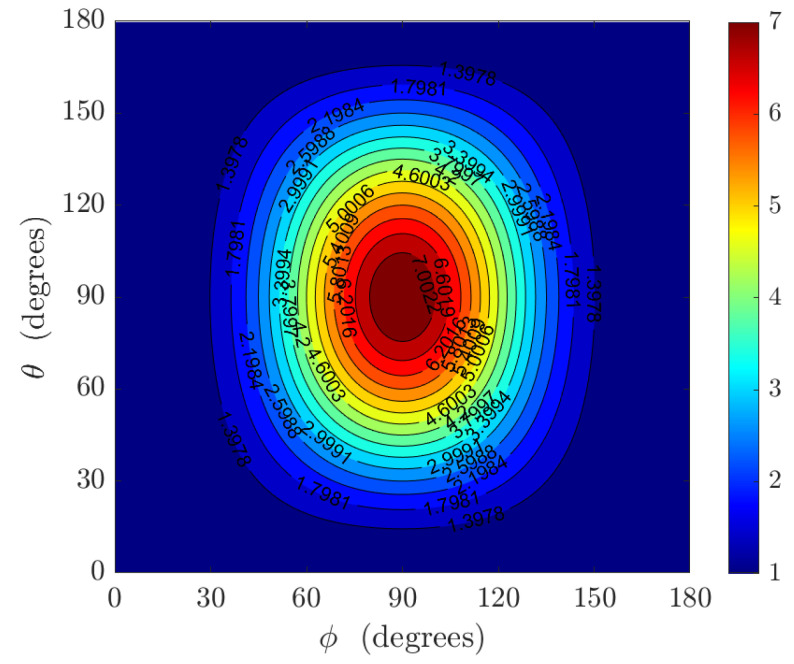
Plot of the performance ratio $r_{\phi }$ versus the polar angle and azimuth angle for $\sigma_{\psi }=0.05$.

To study the ratio of the HCRB of the skewed biaxial vector sensor to the perfectly orthogonal case, we define the performance ratio

(37) $$\begin{array}{ccc} r_{\phi } & := & \frac{{\mathrm{HRCB}}_{\phi }}{{\left.{\mathrm{HCRB}}_{\phi }\right|}_{\sigma_{\psi }=0}} \end{array}$$

such that $r_{\phi }>1$ indicates degraded performance, $r_{\phi }<1$ implies improved performance, and $r_{\phi }=1$ indicates equal performance. Plot of the performance ratio $r_{\phi }$ versus the direction of arrival is shown in Figure 7.

The maximum degradation occurs at azimuth angles of 90${}^{\circ }$ and 270${}^{\circ }$. This trend is also noticed in Figure 6b, where the bound for the polar angle is maximum at $90^{\circ }$ and $270^{\circ }$. The skew angle standard deviation $\sigma_{\psi }=0.05$ gives a maximum performance ratio of approximately 7.

### 4.3. Hybrid CRB for the Skew Angle, HCRB${}_{\psi }$

**Figure 8:**
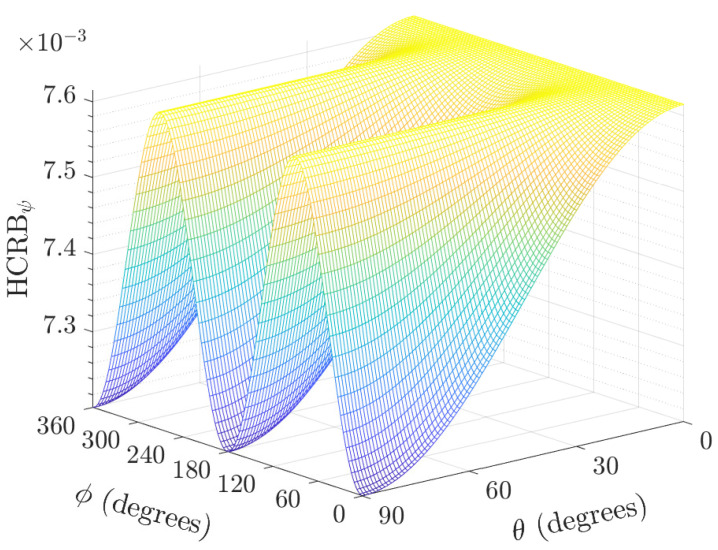
Plot of ${\mathrm{HCRB}}_{\psi }$ versus the direction-of-arrival polar and azimuth angles for K=100 samples, $\sigma_{s}^{2}/\sigma_{n}^{2}=5$, and $\sigma_{v}=0.0873\;\mathrm{rad}$.

In applications where the skew angle is to be estimated in order to implement some corrections in beamforming solutions, as studied in [15,26], the hybrid Cramér-Rao bound derived as (33) gives the estimation variance lower bound as a function of the direction of arrival, signal-to-noise ratio, and the number of samples. As the SNR tends to zero, the bound tends to $\sigma_{\psi }^{2}$, implying that the data provide little or no information concerning the biaxial sensor geometry. The plot of the ${\mathrm{HCRB}}_{\psi }$ versus the directions-of-arrival for the polar and azimuth angles are shown in Figure 8.

The ${\mathrm{HCRB}}_{\psi }$ varies along both the polar angle and azimuth angle. The best variance occurs at $\theta =90^{\circ }$ but for only $\phi =0^{\circ }$ and $180^{\circ }$. This implies that, to more accurately estimate the skew angle (for calibration or post-recording corrections), the source needs to be incident from $\phi =0^{\circ }$ and $180^{\circ }$. Generally, the ${\mathrm{HCRB}}_{\psi }$ increases as $\sigma_{\psi }^{2}$ increases. This implies that the more the skew angle varies, the less accurately it can be estimated. The worst ${\mathrm{HCRB}}_{\psi }$ is equal to the skew angle variance as expected. This is analytically obtained by maximizing ${\mathrm{HCRB}}_{\psi }$ with respect to the azimuth and polar angles.

## 5. Hybrid Maximum Likelihood and Maximum A Posterior (Hybrid ML/MAP) Estimator to Verify the Derived Bounds

In this section, the hybrid ML/MAP or joint MAP and ML estimator (JMAPMLE) is used to verify the derived HRCB for the direction of arrival polar and azimuth angles [40] (p. 12). The statistical data model of the posterior probability density function (PDF) is given as

(38) $$\begin{array}{c} \;p\left(\theta ,\phi ,\psi |\tilde{z}\right)\propto p\left(\tilde{z},\psi |\theta ,\phi \right)\\ =p\left(\tilde{z}|\psi ,\theta ,\phi \right)p\left(\psi \right) \end{array}$$

where

(39) $$\begin{array}{ccc} p(\tilde{z}|\psi ,\theta ,\phi ) & = & \frac{1}{\pi \left|\Gamma \right|}\exp\left\{-{\left[\tilde{z}-s⊗{\tilde{a}}_{\psi }\right]}^{H}\Gamma^{-1}\left[\tilde{z}-s⊗{\tilde{a}}_{\psi }\right]\right\} \end{array}$$

is the conditional PDF of the received data $\tilde{z}$. Recall that from (7), $\Gamma =\sigma_{n}^{2}I_{3K}$. The prior PDF of the skew angle ψ is given as

(40) $$\begin{array}{ccc} p(\psi ) & = & \frac{1}{\sqrt{2\pi \sigma_{\psi }^{2}}}\exp\left\{-\frac{\psi^{2}}{2\sigma_{\psi }^{2}}\right\} \end{array}$$

where all variables are as previously defined. The hybrid maximum likelihood-maximum a posterior (hybrid ML/MAP) estimator is, thus,

(41) $$\begin{array}{cc} \left({\hat{\theta }}_{\mathrm{MLE}},{\hat{\phi }}_{\mathrm{MLE}},{\hat{\psi }}_{\mathrm{MAP}}\right) & =\arg\max_{\left(\theta ,\phi ,\psi \right)}\ln p\left(\tilde{z}|\theta ,\phi ,\psi \right)p\left(\psi \right)\\ & \equiv \arg\min_{\left(\theta ,\phi ,\psi \right)}\left\{\frac{1}{\sigma_{n}^{2}}{\left[\tilde{z}-s⊗{\tilde{a}}_{\psi }\right]}^{H}\left[\tilde{z}-s⊗{\tilde{a}}_{\psi }\right]+\frac{\psi^{2}}{2\sigma_{\psi }^{2}}\right\} \end{array}$$

**Figure 9:**
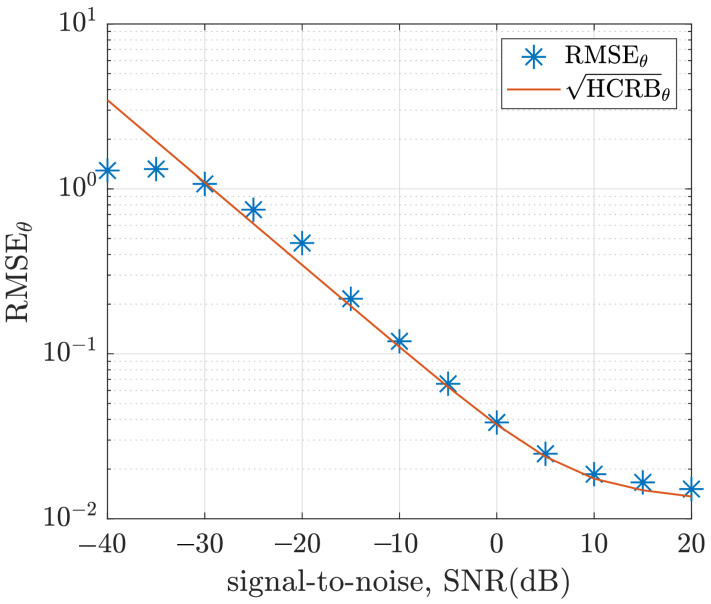
Plot of ${\mathrm{RMSE}}_{\theta }$ and $\sqrt{{\mathrm{HCRB}}_{\theta }}$ versus signal-to-noise ratio for $\left(\theta ,\phi \right)=\left(35^{\circ },30^{\circ }\right)$, $\sigma_{\psi }=0.05$ and K=256 samples.

**Figure 10:**
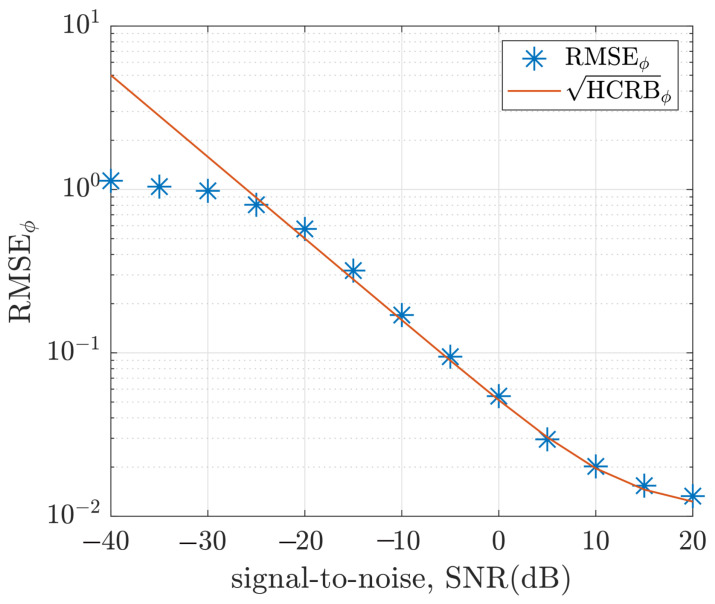
Plot of ${\mathrm{RMSE}}_{\phi }$ and $\sqrt{{\mathrm{HCRB}}_{\phi }}$ versus signal-to-noise ratio for $\left(\theta ,\phi \right)=\left(35^{\circ },30^{\circ }\right)$, $\sigma_{\psi }=0.05$ and K=256 samples.

The Monte Carlo simulation of the hybrid ML/MAP estimator was carried out for L=1000 iterations and K=256 samples. The root mean square of the estimation error and the root HCRB are plotted against the signal-to-noise ratio in Figure 9 for the polar angle, θ, and in Figure 10 for the azimuth angle, ϕ. Each point (on the plots) represents the root-mean-square (RMSE) of the error from L=1000 Monte Carlo iterations and, defined as

(42) $$\begin{array}{ccc} {\mathrm{RMSE}}_{\theta } & = & \sqrt{\frac{1}{L}\sum_{\ell =1}^{L}{\left({\hat{\theta }}_{\mathrm{MLE}}-\theta \right)}^{2}} \end{array}$$

(43) $$\begin{array}{ccc} {\mathrm{RMSE}}_{\phi } & = & \sqrt{\frac{1}{L}\sum_{\ell =1}^{L}{\left({\hat{\phi }}_{\mathrm{MLE}}-\phi \right)}^{2}}. \end{array}$$

Figure 9 and Figure 10 show that the RMSE is close to the derived hybrid Cramér-Rao bounds for a higher signal-to-noise ratio. As the signal-to-noise ratio decreases further, the threshold effect makes it impossible for the RMSE to be close to the derived HCRB.

## 6. Conclusions

The hybrid Cramér-Rao bound of the polar angle, azimuth angle, and skew angle of the non-perpendicular biaxial velocity sensor have been derived in closed form. The angle of skew was modeled as a zero-mean Gaussian random variable with a known variance. It was assumed that all parameters are known, but the three under consideration (emitter’s polar and azimuth angles, and array’s skew angle), and the incident signal are pure tone complex exponentials.

The analysis shows how the hybrid Cramér-Rao bound of the parameters of interest depends on the skew angle variance of the non-perpendicular biaxial velocity sensor. Simply put, the skewness introduces variations of the HCRBs of the polar angle and azimuth angle with the azimuth angle. A comparison of the lower bounds shows that the skewness increases the estimation error in lower bounds. For applications in which the skew angle needs to be estimated, the analysis carried out in this paper shows that the HCRB of the skew angle is bounded upwards by the variance of the skew angle. The hybrid ML/MAP estimator was finally used to demonstrate the correctness of the derived bounds. The generalization of the present study to the case where the signal amplitude is unknown will be left for future research.

## Data Availability

Not applicable.

## Appendix A. Expectations of Sine and Cosine of the Gaussian Random Variable

If $Y:=\left(\psi +\phi \right)∼N\left(\phi ,\sigma_{\psi }^{2}\right)$, then

(A1) $$\begin{array}{ccc} E[\cos(Y)] & = & e^{-{\frac{1}{2}}^{\sigma_{\psi }^{2}}}\cos\left(\phi \right) \end{array}$$

(A2) $$\begin{array}{ccc} E[\sin(Y)] & = & e^{-{\frac{1}{2}}^{\sigma_{\psi }^{2}}}\sin\left(\phi \right) \end{array}$$

**Proof.** If Y:=ψ+ϕ is a Gaussian random variable with mean ϕ and variance $\sigma_{\psi }^{2}$, its moment generating function is given as [41]

(A3) $$\begin{array}{c} E\left[e^{tY}\right]=e^{\phi t}e^{\frac{1}{2}\sigma_{\psi }^{2}t^{2}} \end{array}$$

Recall that

(A4) $$\begin{array}{ccc} E[e^{jY}] & = & E[\cos(Y)+j\sin(Y)] \end{array}$$

which implies that $E\left[\cos\left(Y\right)\right]=ℜ\left\{E\left[e^{jY}\right]\right\}$ and $E\left[\sin\left(Y\right)\right]=ℑ\left\{E\left[e^{jY}\right]\right\}$. Substituting *t* in (A3) with *j*,

(A5) $$\begin{array}{ccc} E[e^{jY}] & = & e^{j\phi }e^{-\frac{1}{2}\sigma_{\psi }^{2}} \end{array}$$

then

(A6) $$\begin{array}{cc} E\left[\cos\left(Y\right)\right] & =ℜ\left\{E\left[e^{jY}\right]\right\}\\ & =e^{-\frac{1}{2}\sigma_{\psi }^{2}}\cos\left(\phi \right) \end{array}$$

and

(A7) $$\begin{array}{cc} E\left[\sin\left(Y\right)\right] & =ℑ\left\{E\left[e^{jY}\right]\right\}\\ & =e^{-\frac{1}{2}\sigma_{\psi }^{2}}\sin\left(\phi \right) \end{array}$$

Expectations of other trig functions of the normal random variable are similarly derived by reducing the trig functions to simple forms. □
